# Melatonin reduces lipid peroxidation and membrane viscosity

**DOI:** 10.3389/fphys.2014.00377

**Published:** 2014-10-06

**Authors:** Russel J. Reiter, Dun-Xian Tan, Annia Galano

**Affiliations:** ^1^Department of Cellular and Structural Biology, UT Health Science CenterSan Antonio, TX, USA; ^2^Departamento de Quimica, Universidad Autonoma Metropolitana – IztapalapaMexico City, Mexico

**Keywords:** polyunsaturated fatty acids, antioxidant cascade, cyclic 3-hydroxymelatonin, AFMK, AMK

## Introduction

Lipid peroxidation (LPO) occurs as a result of the oxidative deterioration of polyunsaturated fatty acids (PUFA), i.e., those that contain two or more carbon–carbon double bonds. The most apparent feature of the oxidative breakdown of lipids is rancidity, a problem that was recognized centuries ago during the storage of fats and oils. Rancidity persists as a widespread problem in today's society because of the common use of polyunsaturated fats and oils.

The outer limiting membrane of cells and membranes of subcellular organelles, e.g., mitochondria, liposomes, peroxisomes, etc., are generally rich in PUFA and their protection from oxidation is essential for the optimal function and survival of the cell. In addition to lipids, cell membranes also contain proteins in varying amounts depending on the unique physiology of the membrane. Thus, the inner mitochondrial membrane, because of its high density of respiratory complex proteins, contains only 20% lipids; this is also the case with chloroplast thylakoid membranes. In contrast, the myelin sheath surrounding axons are up to 80% lipid. Due to the differences in the percentage of lipids in membranes, they are subjected to different degrees of peroxidation.

Membranes are fluid structures and optimal membrane fluidity is required for their proper function. When membrane fatty acids are oxidized, cell membranes become viscous (more rigid). Many factors contribute to the oxidation of membrane lipids and, during aging, cell membranes become progressively more rancid and rigid; this contributes to the degenerative signs of aging.

**Figure 1:**
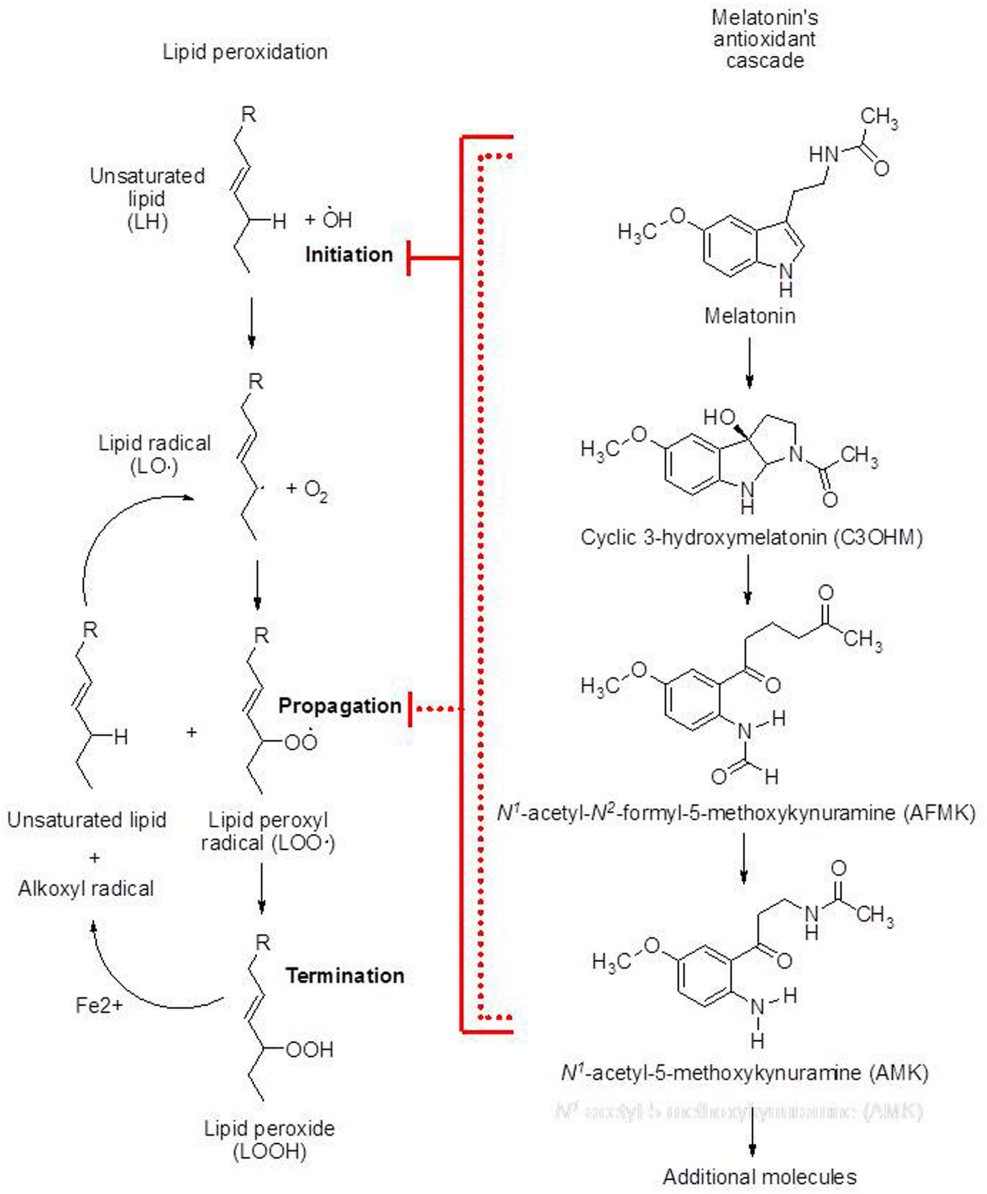
**Schematic representations of lipid peroxidation and melatonin's antioxidant cascade**. The metabolites of melatonin, i.e., c3OHM, AFMK, and AMK, are generated when the preceding molecule in the cascade functions in the detoxification of reactive oxygen or reactive nitrogen species. Melatonin and each of its metabolites reduce the initiation of lipid peroxidation by scavenging the initiating agents, e.g., •OH, ONOO^−^, etc. Additionally the parent molecule and all of its metabolites also interrupt the propagation of lipid peroxidation by scavenging the peroxyl radical. Especially *in vivo*, melatonin effectively protects lipids from peroxidation thereby preventing rancidity and preserving optimal membrane fluidity.

The oxidation of lipids is a highly complex process that is initiated when a hydrogen atom is abstracted from a methylene (–CH_2_–) group by a free radical (Figure 1). PUFA are particularly susceptible to peroxidative initiation because of their numerous carbon–carbon double bonds. Of the free radicals and other reactive oxygen (ROS) and reactive nitrogen species (RNS) generated within cells, the hydroxyl radical (•OH) is easily capable of initiating LPO. In contrast, the superoxide anion radical (O_2_•–) is not sufficiently reactive to abstract a hydrogen atom from a lipid molecule. As a consequence of the initiation of lipid breakdown, a lipid peroxyl radical (ROO•) is eventually generated. ROO• are highly reactive and are capable of abstracting a hydrogen atom from a neighboring lipid (causing another initiation event). This is referred to as the propagation phase of LPO. Due to this auto-oxidative chain reaction, a single initiation event could theoretically lead to the oxidation of all lipids in a cellular organelle, or in a cell. Other reactive species which initiate LPO include peroxynitrite anion (ONOO^−^) and singlet oxygen (^1^O_2_). Because of the highly destructive structural and functional nature of LPO, there is great interest in identifying molecules which reduce the initiation and/or progression of the denaturation of PUFA.

## Melatonin and its derivatives as antioxidants

What has come to be known as melatonin's antioxidant cascade accounts, presumably in large part, for its ability to reduce oxidative damage, including that to PUFA (Tan et al., 2007). When melatonin functions in the detoxification of radicals, the metabolites that are formed are also radical scavengers. The initial derivative that is produced is cyclic 3-hydroxymelatonin (c3OHM) (Figure 1). This derivative functions as a radical scavenger to generate N1-acetyl-N2-formyl-5-methoxykynuramine (AFMK) which, like its predecessor, neutralizes toxic ROS/RNS. In doing so AFMK is metabolized to N1-acetyl-5-methoxykynuramine (AMK). AMK likewise is capable of defeating radicals and beyond this there may yet be other derivatives that function as antioxidants.

Via this cascade of reactions, each molecule of melatonin is predicted to scavenge up to 10 ROS/RNS. This unique property of melatonin makes it highly effective in combatting oxidative stress and LPO. Melatonin is produced in many cells and its synthesis may be upregulated under conditions that elevate oxidative stress in mammals, as happens in plants (Arnao and Hernandez-Ruiz, 2013).

Besides its direct actions as a scavenger, melatonin also stimulates the activities of a variety of antioxidative enzymes including manganese and copper-zinc superoxide dismutase, glutathione peroxidase and reductase and glutamylcysteine ligase (Rodriguez et al., 2004). The combined actions as well as its function at the inner mitochondrial membrane where it limits electron leakage (called radical avoidance) makes melatonin exceptionally effective in reducing oxidative stress.

## Melatonin: reducing lipid peroxidation

The ability of melatonin to protect against LPO has been repeatedly documented in many animal and plant tissues under numerous oxidizing conditions, e.g., ionizing radiation, heavy metal toxicity, drug metabolism, intense exercise, etc. (Garcia et al., 2014). The precise mechanisms by which melatonin and/or its metabolites function to limit LPO is not yet established. The mechanistic information that is available has come primarily from studies on lipid vesicles (micells) containing with one or several phospholipids. Data indicate that melatonin is embedded preferentially in a superficial location in membrane lipid layers near the polar heads of these molecules (Ceraulo et al., 1999). Its juxtaposition to the lipid molecules allows melatonin to protect them from the onslaught of free radicals. The lipid protective actions of melatonin have been proven, both *in vitro* and *in vivo*, and in models of numerous diseases. Among subcellular organelles, membranes and mitochondria have the highest intrinsic levels of melatonin and these concentrations are not diminished when blood levels of the indoleamine are depleted (Venegas et al., 2012). Its small molecular size and its amphiphilic properties facilitate melatonin's penetration into subcellular compartments.

While melatonin reduces the initiation of LPO, according to Marshall et al. (1996), it is not considered to be highly effective as a chain breaking antioxidant. This finding is disputed, however, by the data of Mekhloufi et al. (2007) and of Marchetti et al. (2011) who observed melatonin is in fact highly efficient as a LOO• scavenger. Recently, c3OHM and AMK were proposed as highly effective LOO• scavengers. Hence, melatonin as well as its metabolites function as chain breaking antioxidants (Figure 1).

In plants, the chloroplast envelope as well as their thylakoids possess a high percentage of PUFA; thus, like mitochondria, they are also readily susceptible to LPO. Moreover, like the inner mitochondrial membrane, the electron transport chain in the thylakoid of chloroplasts leak electrons on to O_2_ to generate radical products. Melatonin has been identified in plants where it functions in protecting against oxidative damage (Arnao and Hernández-Ruiz, 2014). The ability of melatonin to protect plant cells from LPO is of special interest since recent data suggests the chloroplasts, like mitochondria, likely produce melatonin (J. Kong et al., unpublished).

Another major contributor to LPO is ONOO–. Like the •OH, it is a powerful initiator of lipid breakdown. Since melatonin also neutralizes the ONOO–, this is another means whereby melatonin may alleviate the decomposition of membrane lipids (Cuzzocrea et al., 1997).

Melatonin also directly scavenges the alkoxyl radical, a product resulting from the transition metal-catalyzed degradation of lipid peroxides (Zavodnik et al., 2006) (Figure 1). This is important for the control of LPO since the alkoxyl radical can abstract a hydrogen atom from a PUFA (Figure 1); the resulting LOO• can obviously continue the propagation of lipid degradation.

## Melatonin's derivatives: reducing lipid peroxidation

Galano et al. (2014) examined the reaction of c3OHM with the •OH and LOO• in both a lipid and aqueous environment by means of Functional Density Theory considering three potential mechanisms of action: radical adduct formation, hydrogen transfer and single electron transfer. Regardless of the polarity of the environment, c3OHM reacted with the •OH at a diffusion controlled rate which was slightly better than that of either melatonin, AFMK or AMK. Against the LOO•, c3OHM was orders of magnitude better than AFMK and AMK and roughly 100-fold better than vitamin E. Although melatonin and its metabolites, AFMK and AMK, are LOO• scavengers, the findings of Galano et al. (2014) indicate that melatonin's ability to resist LPO may also involve its metabolite, c3OHM.

A more direct approach to test c3OHM as a scavenger was taken by Tan et al. (2014). Their results support the conclusion that c3OHM is a highly effective radical scavenger and has particularly high efficacy in protecting molecules that contain haemprotein, e.g., hemoglobin and cytochrome c, from degradation; c3OHM functions by donating a single electron thereby recovering oxidized horseradish peroxidase to its ground state. c3OHM was also found to be a better scavenger of the •OH than vitamin C in recovering oxidized horseradish peroxidase.

Although AFMK provides protection against oxidative stress, there are few studies related to its ability to limit LPO (Galano et al., 2013). AFMK reduces free radical damage to lipids in rat liver homogenates (Tan et al., 2001) while in an *in vivo* study, Manda et al. (2007) reported that the oxidative modification of brain lipids was reduced when mice were treated with melatonin prior to their exposure to ionizing radiation.

Cyclic voltammetry studies found that AFMK is capable of donating two electrons as evidence of its reductive potential. With the aid of electron spin resonance spectroscopy, it was shown that AFMK readily scavenges the •OH which could account for its ability to control LPO. The high efficiency by which AFMK neutralizes the •OH has been confirmed while the data related to the ability of this melatonin metabolite to directly detoxify the LOO• is more limited (Galano, 2011).

While AMK reduces LPO, whether this is due to its direct scavenging ability or a result of its stimulation of antioxidant enzymes has not been determined (Ressmeyer et al., 2003). AMK reportedly scavenges singlet oxygen (^1^O_2_) and nitric oxide (•NO) (Schaffer and Hardeland, 2009). ^1^O_2_ can directly react with carbon–carbon lipid double bonds to yield peroxides (Xia et al., 2012). Besides scavenging •NO, melatonin also inhibits its production (Leon et al., 2006), both of which would control LPO, since •NO couples with O_2_•– to produce ONOO^−^, a proven initiator of LPO.

## Melatonin and membrane fluidity

Since the degree of lipid breakdown in cell membranes generally correlates with the fluidity of these organelles, it is predicted that melatonin would also reduce membrane rigidity. This has been amply demonstrated. Such findings have significant functional relevance, since limiting the movement of molecules in cell membranes, which increased viscosity does do negatively impacts cellular physiology. Aging is characteristically associated with elevated cell membrane rigidity.

Depressed levels of melatonin naturally occur with aging or as a consequence of pinealectomy leads to elevated and levels of LPO and more viscous cellular membranes (Reiter et al., 1999; Hardeland, 2013). Likewise, treatment of senescence-accelerated prone mice (SAMP8) with melatonin preserves mitochondrial membranes in a more fluid state (Garcia et al., 2014). Membrane fluidity relates to the degree of LPO; thus, when the fluidity of membranes is reduced, the amount of oxidized lipids in membranes is increased.

## Concluding remarks

Melatonin is a highly evolutionarily conserved molecule that both directly and indirectly markedly reduces the breakdown of lipids in both animals and plants, especially *in vivo*. What is difficult to determine is whether this protective action is exclusively attributable to its radical scavenging ability or whether it is a consequence of this action by its metabolites c3OHM, AFMK or AMK. It has been difficult to unravel the mechanisms behind melatonin's LPO inhibitory effects since all the metabolites mentioned are formed during melatonin's antioxidant cascade. Finally, melatonin is a well-known stimulator of antioxidative enzymes which would indirectly reduce LPO. Currently, what is known is that both endogenously-generated and exogenously-administered melatonin has an important role in restricting lipid rancidity and preserving optimal membrane fluidity. Also of importance is that neither melatonin nor its metabolites have revealed any pro-oxidant activity in normal cells, a feature that occurs with some classic antioxidants.

### Conflict of interest statement

The Reviewer, Andrzej T Slominski, declares that despite having collaborated with the author Russel J Reiter, the review process was handled objectively and no conflict of interest exists. The authors declare that the research was conducted in the absence of any commercial or financial relationships that could be construed as a potential conflict of interest.
